# Rectal indomethacin alone versus indomethacin and prophylactic pancreatic stent placement for preventing pancreatitis after ERCP: study protocol for a randomized controlled trial

**DOI:** 10.1186/s13063-016-1251-2

**Published:** 2016-03-03

**Authors:** B. Joseph Elmunzer, Jose Serrano, Amitabh Chak, Steven A. Edmundowicz, Georgios I. Papachristou, James M. Scheiman, Vikesh K. Singh, Shyam Varadurajulu, John J. Vargo, Field F. Willingham, Todd H. Baron, Gregory A. Coté, Joseph Romagnuolo, April Wood-Williams, Emily K. Depue, Rebecca L. Spitzer, Cathie Spino, Lydia D. Foster, Valerie Durkalski, on behalf of the SVI study group and the United States Cooperative for Outcomes Research in Endoscopy (USCORE)

**Affiliations:** Division of Gastroenterology and Hepatology, Medical University of South Carolina, MSC 702, 114 Doughty St., Suite 249, Charleston, SC 29425 USA; Institute of Diabetes and Digestive and Kidney Diseases, National Institutes of Health, Bethesda, MD USA; Division of Gastroenterology, University Hospitals Case Medical Center, Cleveland, OH USA; Division of Gastroenterology, Washington University School of Medicine, St Louis, MO USA; Division of Gastroenterology, Hepatology, and Nutrition, University of Pittsburgh Medical Center, Pittsburgh, PA USA; Division of Gastroenterology, University of Michigan Medical Center, Ann Arbor, MI USA; Division of Gastroenterology, Johns Hopkins Medical Institutions, Baltimore, MD USA; Center for Interventional Endoscopy, Florida Hospital, Orlando, FL USA; Department of Gastroenterology and Hepatology, The Cleveland Clinic Foundation, Cleveland, OH USA; Division of Digestive Diseases, Emory University School of Medicine, Atlanta, GA USA; Division of Gastroenterology and Hepatology, University of North Carolina School of Medicine, Chapel Hill, NC USA; Tidelands Health, Murrels Inlet, SC USA; Department of Public Health, University of Michigan Medical School, Ann Arbor, MI USA; Data Coordination Unit, Department of Public Health Sciences, Medical University of South Carolina, Charleston, SC USA

## Abstract

**Background:**

The combination of prophylactic pancreatic stent placement (PSP) – a temporary plastic stent placed in the pancreatic duct – and rectal non-steroidal anti-inflammatory drugs (NSAIDs) is recommended for preventing post-endoscopic retrograde cholangiopancreatography (ERCP) pancreatitis (PEP) in high-risk cases. Preliminary data, however, suggest that PSP may be unnecessary if rectal NSAIDs are administered. Given the costs and potential risks of PSP, we aim to determine whether rectal indomethacin obviates the need for pancreatic stent placement in patients undergoing high-risk ERCP.

**Methods/Design:**

The SVI (Stent vs. Indomethacin) trial is a comparative effectiveness, multicenter, randomized, double-blind, non-inferiority study of rectal indomethacin alone versus the combination of rectal indomethacin and PSP for preventing PEP in high-risk cases. One thousand four hundred and thirty subjects undergoing high-risk ERCP, in whom PSP is planned solely for PEP prevention, will be randomized to indomethacin alone or combination therapy. Those who are aware of study group assignment, including the endoscopist, will not be involved in the post-procedure care of the patient for at least 48 hours. Subjects will be assessed for PEP and its severity by a panel of independent and blinded adjudicators. Indomethacin alone will be declared non-inferior to combination therapy if the two-sided 95 % upper confidence bound of the treatment difference is less than 5 % between the two groups. Biological specimens will be obtained from trial participants and centrally banked.

**Discussion:**

The SVI trial is designed to determine whether PSP remains necessary in the era of NSAIDs pharmacoprevention. The associated bio-repository will establish the groundwork for important scientific breakthrough.

**Trial registration:**

NCT02476279, registered June 2015.

## Background

Based on clinical trial data, the combination of prophylactic pancreatic stent placement (PSP) – a temporary plastic stent placed in the pancreatic duct – and rectal non-steroidal anti-inflammatory drug (NSAID) administration is recommended for preventing post-endoscopic retrograde cholangiopancreatography (ERCP) pancreatitis (PEP) in high-risk cases [1, 2]. PSP, however, is technically challenging, time-consuming, costly, and potentially dangerous [3–6]. Moreover, randomized controlled trials (RCTs) demonstrating the efficacy of stent placement were un-blinded in design and conducted by expert endoscopists who are highly skilled in pancreatic endotherapy, potentially exaggerating the benefits of this intervention.

Secondary analysis of a large-scale multicenter RCT of rectal indomethacin for preventing PEP in high-risk cases suggested that subjects who received indomethacin alone were less likely to develop PEP than those who received a pancreatic stent alone or the combination of indomethacin and stent, even after adjusting for underlying differences in subject risk [7]. In addition, a recent network meta-analysis comparing the data supporting PSP with those supporting prophylactic NSAIDs demonstrated that rectal NSAIDs alone are not inferior to combination therapy [8]. This observation is biologically plausible because a strategy of indomethacin alone avoids manipulation of the pancreatic orifice and instrumentation of the pancreatic duct – interventions necessary to place a stent but also known to contribute to the risk of PEP.

Given the costs and potential disadvantages of PSP, minimizing its use in ERCP practice could result in major clinical and economic benefits. Therefore, our aim is to determine whether rectal indomethacin obviates the need for PSP in patients undergoing high-risk ERCP.

## Methods

### Design

The Stent vs. Indomethacin (SVI) trial is a multicenter randomized controlled trial designed to assess whether rectal indomethacin alone is non-inferior by a pre-specified amount to the combination of rectal indomethacin and PSP for preventing PEP in high-risk cases. The study will take place at nine academic medical centers in the United States. The Medical University of South Carolina will serve as the statistical, data, and clinical coordinating center as well as a clinical site. The remaining eight medical centers will serve as clinical sites that enroll and follow study subjects. Ethical approval has been obtained from the Institutional Review Boards of the Medical University of South Carolina, Emory University, Johns Hopkins University, University of Pittsburgh, University of Michigan, Case Western Reserve University, University of Colorado, Washington University, and the Florida Hospital.

### Patients

The eligibility criteria are listed in Table 1. Informed consent will be obtained from all study participants. We plan to enroll adult patients at elevated risk for PEP who require pancreatic stent placement for the sole purpose of pancreatitis prevention. Patients will be considered to be at high risk for PEP if their indication for PSP is one of the following criteria: prior PEP, clinical suspicion of sphincter of Oddi dysfunction, pancreatic sphincterotomy, precut sphincterotomy, difficult cannulation, or short-duration pneumatic dilatation of an intact biliary sphincter. Patients may also be included if they meet two or more of the following criteria: age less than 50 years *and* female sex, a history of recurrent pancreatitis (at least two episodes), three or more injections of contrast into the pancreatic duct, pancreatic acinarization, or pancreatic brush cytology. Patients will be excluded if they have standard contraindications to ERCP, have a contraindication to the use of NSAIDs (e.g., allergy, known renal failure, or active peptic ulcer disease), have experienced recent acute pancreatitis, or have an anticipated low risk of PEP (e.g., those with chronic calcific pancreatitis, a pancreatic head mass, or those undergoing biliary interventions through a pre-existing sphincterotomy).

**Table 1:** Eligibility criteria

Any patient undergoing endoscopic retrograde cholangiopancreatography (ERCP) in whom pancreatic stent placement is planned for post-ERCP pancreatitis prevention, is at least 18 years old, provides informed consent, *and*:	
Has one of the following criteria:	*Or* two of the following criteria:
Clinical suspicion of sphincter of Oddi dysfunction	Age under 50 years old and of female gender
History of post-ERCP pancreatitis	History of recurrent pancreatitis
Pancreatic sphincterotomy	At least 3 pancreatic injections, with at least 1 injection to tail
Pre-cut (access) sphincterotomy	Pancreatic acinarization
Difficult cannulation	Pancreatic brush cytology
Short duration (≤1 min) balloon dilation of intact biliary sphincter	
Exclusion criteria	
Ampullectomy	
Case in which a pancreatic stent is placed for therapeutic intent	
Unwillingness or inability to consent for the study	
Pregnancy	
Breastfeeding mother	
Standard contraindications to ERCP	
Allergy to aspirin or NSAIDs	
Known renal failure (creatinine >1.4 mg/dl)	
Ongoing or recent (within 2 weeks) hospitalization for gastrointestinal hemorrhage	
Ongoing or recent (within 1 week) hospitalization for acute pancreatitis	
Known chronic calcific pancreatitis	
Pancreatic head mass	
Procedure performed on major papilla/ventral pancreatic duct in patient with pancreas divisum (no manipulation of minor papilla)	
ERCP for biliary stent removal or exchange without anticipated pancreatogram	
Subjects with prior biliary sphincterotomy now scheduled for repeat biliary therapy without anticipated pancreatogram	
Anticipated inability to follow protocol	
Absence of rectum	

*NSAIDs* non-steroidal anti-inflammatory drugs

### Procedure and interventions

All ERCP-related interventions with the exception of stent placement will be dictated by the performing endoscopist. During the procedure, if an inclusion criterion has been met *and* none of the exclusion criteria are present *and* the papilla is accessible, the subject will be randomized to receive 100 mg of rectal indomethacin (two 50 mg suppositories) only or the combination of prophylactic stent and 100 mg of rectal indomethacin. Randomization will occur in a 1:1 fashion using a web-based central randomization system that will ensure treatment balance within site.

Indomethacin suppositories will be administered in all subjects by an endoscopy nurse, technician, or the endoscopist immediately after completion of the ERCP while the patient is still in the endoscopy suite. The technique by which prophylactic pancreatic stents are placed, and the type of stent used, will not be directed by the study protocol but rather deferred to the judgment of the endoscopist. This approach is intended to mimic real-world practice, wherein variations in stent type, caliber, and length exist [9]. If a pancreatic stent is placed to facilitate biliary access in a patient randomized to the indomethacin alone arm, it will be removed before the end of the case. Since intravenous fluid (IVF) type and rate may influence the development of the primary and secondary endpoints [10, 11], all decisions regarding IVF administration made by the (unblinded) endoscopy team will be implemented prior to randomization. Subsequently, decisions pertaining to IVF administration will be made by blinded clinical personnel.

Since the endoscopist(s), endoscopy nurse, and technician/assistant involved in the ERCP will be aware of whether or not a stent was placed, these individuals will not be involved in the post-procedure care of the patient for at least 48 hours after the procedure, at which point the presence or absence of the primary endpoint (PEP) will have become apparent. This approach is critical to maintaining blinding (of patients, treating clinical personnel, and outcome adjudicators), which ensures equal co-interventions between study groups and unbiased adjudication of the primary outcome. Additionally, the endoscopy report and medical record will not state whether a stent was placed. If the patient receives a stent, they will be contacted 1–2 weeks after the ERCP to arrange an abdominal radiograph to ensure spontaneous passage of the pancreatic stent.

Participating endoscopists will have the option of implementing a provider-specific post-procedure order set that is activated prior to randomization and executed uniformly regardless of a subject’s study group assignment. This order set may include IVF, analgesic, and antiemetic administration as well as parameters for hospital admission. If an order set is not available or has not been activated, a designating blinded clinician will oversee the post-procedure care of the patient. If a subject is hospitalized, clinical decisions will be made by an inpatient team which does not include the endoscopist (or other unblinded personnel) for at least 48 hours after the ERCP.

### Follow-up

Subjects will be contacted 5 and 30 days after the ERCP. The goal of the first follow-up is to ascertain data necessary to adjudicate the primary endpoint. The goal of the second follow-up is to ascertain data necessary to adjudicate the secondary outcome and assess for delayed serious adverse events.

### Outcomes

The primary outcome is PEP and the secondary outcome is the severity of PEP. Using the consensus definition of PEP as a diagnostic framework [12], three adjudicators will independently assess study outcomes based on review of a site-provided adverse event narrative and de-identified medical records for each study subject hospitalized within 2 days after the ERCP. In order to ensure blinding of the adjudicators, all information pertaining to radiographic studies and prophylactic stent placement will be redacted from the medical records.

### Statistical considerations

Given the high economic and opportunity costs associated with pancreatic stent placement, as well as the risks of attempted but unsuccessful insertion, we believe that rectal indomethacin alone would become the dominant strategy in clinical practice if we can demonstrate that it results in less than a 5 % greater absolute PEP rate compared to the combination of indomethacin and PSP. This non-inferiority margin is based on a combination of statistical reasoning and clinical judgment and was chosen to ensure that the overall PEP proportion of the new treatment (indomethacin alone) demonstrates a clinically *unimportant* difference from the active comparator arm (the combination of stent and indomethacin) as well as a clinically relevant superiority over a putative placebo (i.e., stent alone).

From a statistical perspective, the non-inferiority margin should retain at least 50 % of the superiority of the combination of stent and indomethacin (the active control in the trial) when compared to stent alone [13, 14]. Our recent indomethacin RCT revealed that the absolute risk difference in the proportion of subjects with PEP between those who received indomethacin plus stent versus those who received stent alone was 6.4 % (95 % CI: 0.5 %, 12.3 %) [1]. Therefore, 50 % of this value provides a non-inferiority margin of 3.2 %. Independent to the statistical approach, a questionnaire was circulated to ERCP experts regarding how much better (in absolute terms) combination therapy would have to be in preventing PEP as compared to indomethacin alone to justify continuing its use in clinical practice. Seven of 11 respondents said that combination therapy would have to be 10 % more effective and the remaining 4 said that it had to be at least 5 % more effective. Based on both the statistical and clinical information, the investigators unanimously agreed upon a non-inferiority margin of 5 %, judging that a difference of greater than 5 % constitutes an important difference in the risk of PEP.

The sample size was estimated using a confidence interval approach focusing on the upper confidence limit for a difference in proportions via simulations using nQuery [15]. Based on the rate of PEP in the combination therapy group in our prior indomethacin RCT [1], we estimated that 1300 subjects (650 per treatment group) are necessary to achieve at least 85 % likelihood of identifying less than a 5 % absolute difference in PEP rates between the two treatment groups. The intention-to-treat (ITT) principle will be applied to the primary analysis and, therefore, to safeguard against an approximate 5 % drop-in/out and missing data rate in the two treatment groups, we increased the sample size by a factor of 1.1 which is derived from 1/(1−*R*)^2^, where R is the proportion of non-adherence [16]. Thus a total of 1430 subjects will be enrolled and randomized.

For the analysis of the primary outcome, the two-sided 95 % upper confidence bound (equivalent to the one-sided 97.5 % upper confidence bound) of the observed risk difference in the proportion of patients developing PEP between the two treatment groups will be calculated. Indomethacin alone will be declared non-inferior to combination therapy if the two-sided 95 % upper confidence bound of the treatment difference is less than 5 %. If indomethacin is found to be non-inferior, an analysis for superiority will be conducted using a one-sided two-sample test for independent binomial proportions.

Two interim analyses for futility using conditional power will be conducted when approximately one third (*N* is approximately 472) and two thirds (*N* is approximately 958) of the total required number of randomized subjects have been evaluated for the primary outcome and all of the outcomes to be used in the analysis are adjudicated. The goal of the interim analysis plan is to determine whether to stop the trial early because it is unlikely to show non-inferiority at the final analysis. A conditional power will be calculated to assess the probability of observing non-inferiority at the final analysis conditional on the observed data and assumptions on the PEP event rates for the remainder of the trial [17].

### Clinical monitoring

A comprehensive site monitoring plan aims to ensure that the trial is conducted in accordance with the approved protocol, regulatory standards, and good clinical practice. Verification of study and regulatory documents will be conducted remotely. Scheduled and “for-cause” on site monitoring visits to each center will be conducted to verify study data and outcomes relative to source documents and to ensure compliance with study procedures.

## Discussion

Prophylactic pancreatic stent placement is thought to reduce the risk of PEP by relieving pancreatic ductal hypertension that develops due to procedure-induced edema and stenosis of the pancreatic orifice [4, 18, 19]. PSP, however, is not completely effective because other pathophysiologic mechanisms, such as chemical, allergic, enzymatic, and infectious injury also contribute to PEP [19] and may be induced or potentiated by the process of placing a pancreatic stent. Indeed, the superiority of indomethacin mono-prevention over any strategy involving PSP is mechanistically plausible because the indomethacin strategy avoids manipulation of the pancreatic orifice and instrumentation of the pancreatic duct.

Indomethacin mono-prevention may offer several additional advantages over combination therapy. First, such a strategy avoids the phenomenon of attempted but failed PSP, which is associated with a high rate of PEP by activating aforementioned pathogenic factors but providing no ductal decompression [6]. It would also eliminate the 4 % of cases that result in significant non-pancreatitis adverse events induced by PSP, such as stent migration and duct perforation [20], as well as the rare adverse events that occur during follow-up upper endoscopy to remove retained stents. It would substantially reduce healthcare expenditures by eliminating the cost of stent placement in most cases, as well as eliminating the need for follow-up abdominal radiography (to ensure spontaneous passage of the stent) and follow-up upper endoscopies to remove retained stents. Indeed, a cost-benefit analysis revealed that a prevention strategy employing rectal indomethacin alone could save approximately US$150 million annually in the United States compared with a strategy of PSP alone, and US$85 million compared with a strategy of indomethacin and PSP [7]. Finally, it would allow broader delivery of endoscopic care (particularly in resource-limited environments) by allowing additional time for other endoscopic procedures and interventions.

The SVI trial is a reappraisal of the effectiveness of PSP in the era of NSAID pharmacoprevention and an opportunity to address the main limitations of prior stent studies – the lack of blinding and the unclear generalizability of the results. The SVI trial will be the first PSP RCT to blind subjects, caregivers, and outcomes assessors. Given the potential impact of co-interventions (IVF, analgesics) on the outcome and the subjective nature of the definition of PEP, blinding in such a trial is necessary to minimize bias in the care of patients and in the adjudication of outcomes. Indeed, the benefit of PSP may have been exaggerated in prior studies because of differential care of patients who have received stents patients (closer observation, more aggressive IVF administration, etc.) or biased interpretation of the definition of PEP based on study group assignment. Furthermore, the large number of participating centers and endoscopists in the SVI trial will allow a broader assessment of the impact of practice and skill variations on the effectiveness of PSP, augmenting the generalizability of the results.

A PSP alone arm is not included in this study because such a strategy is unlikely to remain clinically viable. Robust clinical trial data confirm the essential role of rectal indomethacin in clinical practice and the European Society of Gastrointestinal Endoscopy guidelines recommend rectal indomethacin or diclofenac to all patients undergoing ERCP as a grade A recommendation [2, 21, 22]. In light of this evidence-base, and considering that indomethacin is very safe and inexpensive, PSP alone no longer plays a role in high-risk patients without contraindications to a single dose of NSAIDs. The time and cost necessary to include a stent alone arm in this RCT were not considered justifiable, and the investigators felt that withholding indomethacin from high-risk study subjects would represent suboptimal care.

A major focus of the SVI trial is to establish a repository of biological specimens from study subjects upon which future translational studies can be conducted. To further explore the pathophysiology of acute pancreatitis, the molecular predictors of severity, the mechanisms by which indomethacin protects against PEP, and other important questions, we will obtain and bank whole blood, serum, plasma, urine, stool, and duodenal fluid from trial participants. The SVI investigators and translational research committee are developing a conceptual framework of translational research initiatives using bio-repository samples, including comparisons of indomethacin levels, conventional measures of inflammation and drug activity, as well as comparisons of genetic, microbial, and host/bacterial metabolomic profiles between subjects who do and do not develop PEP.

## Summary

The SVI trial is a natural next step in the advancement of our understanding of PEP prevention by answering a critically important clinical question: *can we replace an invasive and costly preventive strategy with a safe and inexpensive one?* Through the conduct of this study we hope to determine whether PSP remains necessary in the era of NSAIDs pharmacoprevention. In the process, we will establish the groundwork for major translational research breakthroughs in the fields of endoscopy and pancreatology.

## Trial status

This randomized controlled trial began enrolling patients in September of 2015.

## Abbreviations

ERCP: endoscopic retrograde cholangiopancreatography
IVF: intravenous fluid
NSAIDs: non-steroidal anti-inflammatory drugs
PEP: post-ERCP pancreatitis
PSP: pancreatic stent placement
RCT: randomized controlled trial
SVI: Stent vs. Indomethacin trial
