# Correction to: Rectal indomethacin alone versus indomethacin and prophylactic pancreatic stent placement for preventing pancreatitis after ERCP: study protocol for a randomized controlled trial

**DOI:** 10.1186/s13063-020-04458-0

**Published:** 2020-06-03

**Authors:** B. Joseph Elmunzer, Jose Serrano, Amitabh Chak, Steven A. Edmundowicz, Georgios I. Papachristou, James M. Scheiman, Vikesh K. Singh, Shyam Varadarajulu, John J. Vargo, Field F. Willingham, Todd H. Baron, Gregory A. Coté, Joseph Romagnuolo, April Wood-Williams, Emily K. Depue, Rebecca L. Spitzer, Cathie Spino, Lydia D. Foster, Valerie Durkalski, on behalf of the SVI study group and the United States Cooperative for Outcomes Research in Endoscopy (USCORE)

**Affiliations:** 1 Division of Gastroenterology and Hepatology, Medical University of South Carolina, MSC 702, 114 Doughty St., Suite 249, Charleston, SC 29425 USA; 2 Institute of Diabetes and Digestive and Kidney Diseases, National Institutes of Health, Bethesda, MD USA; 3 Division of Gastroenterology, University Hospitals Case Medical Center, Cleveland, OH USA; 4 Division of Gastroenterology, Washington University School of Medicine, St Louis, MO USA; 5 Division of Gastroenterology, Hepatology, and Nutrition, University of Pittsburgh Medical Center, Pittsburgh, PA USA; 6 Division of Gastroenterology, University of Michigan Medical Center, Ann Arbor, MI USA; 7 Division of Gastroenterology, Johns Hopkins Medical Institutions, Baltimore, MD USA; 8 Center for Interventional Endoscopy, Florida Hospital, Orlando, FL USA; 9 Department of Gastroenterology and Hepatology, The Cleveland Clinic Foundation, Cleveland, OH USA; 10 Division of Digestive Diseases, Emory University School of Medicine, Atlanta, GA USA; 11 Division of Gastroenterology and Hepatology, University of North Carolina School of Medicine, Chapel Hill, NC USA; 12 Tidelands Health, Murrels Inlet, SC USA; 13 Department of Public Health, University of Michigan Medical School, Ann Arbor, MI USA; 14 Data Coordination Unit, Department of Public Health Sciences, Medical University of South Carolina, Charleston, SC USA

**Correction to: Trials (2016) 17:120**

**https://doi.org/10.1186/s13063-016-1251-2**

Following publication of the original article [1], the authors identified an error in the author name of Shyam Varadarajulu.

The incorrect author name is: Shyam Varadurajulu

The correct author name is: Shyam Varadarajulu
